# Evaluating the impact of the diagnosis-related groups payment system on laparoscopic uterine fibroid surgery outcomes: insights from a single-center study

**DOI:** 10.3389/fpubh.2025.1555444

**Published:** 2025-06-10

**Authors:** Jingjing Lu, Yiming Pan, Lu Li, Lili Chu, Yanshan Jin

**Affiliations:** ^1^Department of Obstetrics and Gynecology, The Affiliated People’s Hospital of Ningbo University, Ningbo, China; ^2^Department of Ultrasonic, The Affiliated People’s Hospital of Ningbo University, Ningbo, China

**Keywords:** diagnosis-related groups, laparoscopic uterine fibroid surgery, healthcare costs, surgical outcomes, hospital resource utilization, payment systems

## Abstract

**Objective:**

This study aimed to assess the impact of implementing the diagnosis-related groups (DRGs) payment system on hospitalization costs and medical service outcomes at a single institution. The objective was to identify effective cost-saving strategies and guide healthcare practices to support the ongoing adoption of the DRGs system.

**Methods:**

This retrospective study included 616 patients, categorized into three groups based on the payment system in effect during their treatment: a 6-month period under fee-for-service (FFS), a 6-month period following the trial implementation of DRGs (TI-DRGs), and a 6-month period after the official implementation of DRGs (OI-DRGs). Each group was further divided into two subgroups according to the surgical intervention received (either laparoscopic myomectomy or laparoscopic hysterectomy). Data collected included total medical costs, examination fees, surgical costs, medication and supply expenses, length of hospital stay, operation time, intraoperative blood loss, incidence of postoperative anemia, and frequency of blood transfusions.

**Results:**

Total medical costs in the OI-DRGs group were 6.6 and 9.0% higher than those in the FFS and TI-DRGs groups, respectively (*p* < 0.001). Examination costs followed a similar pattern, with the OI-DRGs group showing increases of 5.3 and 12.3% compared to the FFS and TI-DRGs groups (*p* < 0.001). Operation costs also varied significantly among the three groups; the OI-DRGs group incurred 17.1 and 10.5% higher costs than the FFS and TI-DRGs groups, respectively (*p* < 0.001). There were no significant differences among the groups in terms of hospital stay duration, operation time, or intraoperative blood loss. In the FFS group, 57 patients developed postoperative anemia and 14 required blood transfusions; in the TI-DRGs group, 52 patients developed anemia and 16 received transfusions; and in the OI-DRGs group, 74 patients developed anemia with 16 requiring transfusions. However, these differences were not statistically significant.

**Conclusion:**

In summary, the implementation of DRGs for laparoscopic uterine leiomyoma surgery did not lead to a significant reduction in total medical costs. Overall costs were influenced by multiple factors, including the DRG phase, length of stay, type of surgery, and the presence of concurrent procedures. The findings from our single-center study differ from the mainstream view, highlighting that the effects of DRG implementation can be highly context-specific, shaped by local policies, hospital practices, and patient case-mix, which may limit the generalizability of these results beyond our institution or region.

## Introduction

For many years, hospital billing has primarily relied on the fee-for-service (FFS) model, which has been widely criticized for incentivizing increased volumes of inpatient services to maximize utilization and revenue (1). However, this model has contributed to the rapid escalation of national healthcare expenditures in many countries (2).

In response, the diagnosis-related groups (DRGs) payment system was introduced in the United States in 1983 for Medicare reimbursement (3). Unlike the cost-based FFS model, DRGs aim to reduce inpatient healthcare costs by incentivizing efficiency and limiting unnecessary services. However, previous studies, particularly those focused on caesarean sections and appendectomies, have reported mixed outcomes regarding the DRG system’s effectiveness in reducing costs and resource utilization (4–7).

In China, the case-mix system was adopted as part of the 2009 national healthcare reform, with pilot programs launched in cities such as Beijing and Shanghai (8). In Zhejiang Province, DRG-based performance management was initiated on a trial basis on October 1, 2021, and officially implemented on December 1, 2023.

Uterine fibroids are the most common benign tumors among women of reproductive age, with an estimated incidence rate of approximately 40% (9). Minimally invasive procedures, such as laparoscopic myomectomy and laparoscopic hysterectomy, are considered preferred surgical treatments due to their reduced morbidity and faster recovery times.

This study aims to compare clinical outcomes and medical costs associated with laparoscopic myomectomy and laparoscopic hysterectomy under FFS and DRGs payment systems. We seek to identify cost-saving strategies and promote practice changes that support the sustained and effective adoption of DRGs in clinical settings.

## Methods

Clinical data were collected from 616 patients who underwent either laparoscopic myomectomy or laparoscopic hysterectomy at the Affiliated People’s Hospital of Ningbo University. Patients were categorized into three groups based on the payment model and treatment period: (1) a 6-month period under the fee-for-service (FFS) model (April 1, 2021 – September 30, 2021); (2) a 6-month period during the trial implementation of diagnosis-related groups (TI-DRGs) (October 1, 2021 – March 31, 2022); and (3) a 6-month period following the official implementation of DRGs (OI-DRGs) (December 1, 2023 – May 31, 2024). Each group was further subdivided by type of surgical procedure, resulting in six subgroups: FFS laparoscopic myomectomy (FFS-LM), FFS laparoscopic hysterectomy (FSS-LH), TI-DRGs laparoscopic myomectomy (TI-DRGs-LM), TI-DRGs laparoscopic hysterectomy (TI-DRGs-LH), OI-DRGs laparoscopic myomectomy (OI-DRGs-LM), and OI-DRGs laparoscopic hysterectomy (OI-DRGs-LH).

This study was approved by the Ethics Review Committee of the Affiliated People’s Hospital of Ningbo University (approval no. 2024-087). To control for confounding variables, there were no changes in surgical staffing, protocols, or institutional policies across the three periods. All laparoscopic procedures were performed by senior gynecologists and their respective teams. Preoperative and postoperative (day 2) complete blood counts were conducted at our institution for all patients.

Inclusion criteria were: (1) clinical diagnosis of uterine fibroids, (2) treatment by laparoscopic surgery, and (3) postoperative pathological confirmation of uterine leiomyoma. Exclusion criteria included: (1) incomplete clinical data, (2) presence of severe hematologic disorders or cardiovascular disease, (3) active infections or malignancies, and (4) concurrent surgeries involving other organ systems.

The following variables were compared across groups: age, length of hospital stay, total medical costs, examination costs, surgical costs, medication costs, supply costs, operative time, intraoperative blood loss, incidence of postoperative anemia, and frequency of blood transfusion.

Statistical analysis was performed using SPSS version 25.0. Categorical variables were analyzed using the Pearson chi-square test. Continuous variables across multiple groups were assessed using one-way analysis of variance (ANOVA), followed by the least significant difference (LSD) test for *post hoc* comparisons. A multiple linear regression model was constructed to identify predictors of total medical costs. Variance Inflation Factor (VIF) diagnostics revealed values ranging from 1.030 to 1.277 across all predictors, establishing their statistical independence (with VIF = 1 representing zero multicollinearity). This finding obviated the need for further collinearity adjustments in our analysis. All *p*-values were two-sided, with values <0.05 considered statistically significant.

## Results

Data were collected across three distinct time periods. There were no statistically significant differences in age distribution among the three groups. During the FFS period, 196 patients were included, with 159 undergoing laparoscopic myomectomy (FFS-LM) and 37 undergoing laparoscopic hysterectomy (FFS-LH). In the TI-DRGs period, 205 patients underwent surgery. Specifically, 159 underwent laparoscopic myomectomy (TI-DRGs-LM) and 46 underwent laparoscopic hysterectomy (TI-DRGs-LH). During the OI-DRGs period, 215 patients were included, with 176 undergoing laparoscopic myomectomy (OI-DRGs-LM) and 39 undergoing laparoscopic hysterectomy (OI-DRGs-LH).

We analyzed total medical costs (including all hospitalization expenses), along with examination costs (laboratory and imaging charges), operation costs (including anesthesia and surgical fees), medication costs, and supply costs. A comparison of these cost variables across the three periods is summarized in Table 1. Significant differences were observed in total medical costs among the FFS, TI-DRGs, and OI-DRGs groups. Total medical costs in the OI-DRGs group were 6.6 and 9.0% higher than in the FFS and TI-DRGs groups, respectively (*p* < 0.001). Examination costs in the OI-DRGs group were also 5.3 and 12.3% higher than those in the FFS and TI-DRGs groups, respectively (*p* < 0.001). Operation costs differed significantly across the three groups, with the OI-DRGs group incurring 17.1 and 10.5% higher costs than the FFS and TI-DRGs groups, respectively (*p* < 0.001). Additionally, supply costs were significantly higher in the FFS and OI-DRGs groups compared to the TI-DRGs group (*p* = 0.001). Subgroup analysis revealed that in the OI-DRGs-LM subgroup, total medical costs, examination costs, and operation costs were significantly higher than those in the FFS-LM and TI-DRGs-LM subgroups (*p* < 0.05). The TI-DRGs-LM subgroup had lower examination, medication, and supply costs compared to the FFS-LM subgroup. For laparoscopic hysterectomy, operation costs in the OI-DRGs-LH group were higher than those in both the TI-DRGs-LH and FFS-LH groups. Notably, among all groups, the OI-DRGs-LH subgroup exhibited the lowest supply costs.

**Table 1:** Descriptive analysis of basic characteristics (continuous variables).

Variable	Group	LM-related subgroup	LH-related subgroup	Number	Average	SD	F	*P*-value
Age (years)	FSS			196	45.57	6.91	0.621	0.538
	TI-DRGs			205	45.95	6.88		
	OI-DRGs			215	45.16	7.75		
		FSS-LM		159	44.35	6.60	1.424	0.242
		TI-DRGs-LM		159	44.45	6.47		
		OI-DRGs-LM		176	43.35	6.84		
			FSS-LH	37	50.78	5.76	2.172	0.118
			TI-DRGs-LH	46	51.11	5.70		
			OI-DRGs-LH	39	53.33	6.25		
Length of stay (days)	FSS			196	5.87	1.54	0.557	0.573
	TI-DRGs			205	5.71	1.38		
	OI-DRGs			215	5.78	1.64		
		FSS-LM		159	5.82	1.52	1.590	0.205
		TI-DRGs-LM		159	5.53			
		OI-DRGs-LM		176	5.74	1.66		
			FSS-LH	37	6.11	1.63	0.737	0.480
			TI-DRGs-LH	46	6.35	1.42		
			OI-DRGs-LH	39	5.95	1.56		
Total medical costs (RMB Yuan)	FSS			196	14140.34	2323.88	16.113	<0.001
	TI-DRGs ①			205	13826.41	2253.81		
	OI-DRGs ①②			215	15077.68	2471.44		
		FSS-LM		159	13697.54	2143.61	13.149	<0.001
		TI-DRGs-LM		159	13297.50	2143.61		
		OI-DRGs-LM ③④		176	14973.45	2559.64		
			FSS-LH	37	16043.18	2120.77	0.580	0.561
			TI-DRGs-LH	46	15854.02	1986.75		
			OI-DRGs-LH	39	15548.03	1986.92		
Examination costs (RMB Yuan)	FSS			196	2738.47	596.46	13.442	<0.001
	TI-DRGs ①			205	2568.59	641.43		
	OI-DRGs ①②			215	2884.21	631.22		
		FSS-LM		159	2723.39	606.99	13.149	<0.001
		TI-DRGs-LM ③		159	2525.06	611.53		
		OI-DRGs-LM ③④		176	2872.92	640.99		
			FSS-LH	37	2803.25	551.98	1.237	0.294
			TI-DRGs-LH	46	2719.05	722.69		
			OI-DRGs-LH	39	2935.20	590.33		
Operation costs (RMB Yuan)	FSS			196	5350.63	1233.78	26.078	<0.001
	TI-DRGs ①			205	5667.97	1301.05		
	OI-DRGs ①②			215	6263.40	1372.38		
		FSS-LM		159	5314.29	1243.38	18.494	<0.001
		TI-DRGs-LM		159	5470.94	1331.59		
		OI-DRGs-LM ③④		176	6139.08	1392.85		
			FSS-LH	37	5506.82	1195.55	14.589	<0.001
			TI-DRGs-LH ⑤	46	6349.04	914.74		
			OI-DRGs-LH ⑤⑥	39	6824.43	1130.32		
Medication costs (RMB Yuan)	FSS			196	2065.19	609.13	2.906	0.055
	TI-DRGs			205	1968.96	469.38		
	OI-DRGs			215	1939.44	559.70		
		FSS-LM		159	2072.60	618.31	22.993	0.051
		TI-DRGs-LM ③		159	1935.39	453.71		
		OI-DRGs-LM ③		176	1947.61	582.58		
			FSS-LH	37	2033.32	575.01	1.394	0.252
			TI-DRGs-LH	46	2085.00	508.20		
			OI-DRGs-LH	39	1902.59	446.59		
Medical supply costs (RMB Yuan)	FSS			196	2007.79	941.79	7.309	0.001
	TI-DRGs ①			205	1726.48	702.74		
	OI-DRGs ②			215	1888.81	540.06		
		FSS-LM		159	1673.12	520.95	27.466	<0.001
		TI-DRGs-LM ③		159	1540.68	453.37		
		OI-DRGs-LM ③④		176	1936.76	519.89		
			FSS-LH	37	3445.99	996.38	38.961	<0.001
			TI-DRGs-LH ⑤	46	2368.72	986.62		
			OI-DRGs-LH ⑤⑥	39	1672.43	582.35		
Operation duration (min)	FSS			196	92.43	36.75	1.179	0.308
	TI-DRGs			205	88.34	33.20		
	OI-DRGs			215	87.21	37.72		
		FSS-LM		159	87.40	31.36	0.297	0.743
		TI-DRGs-LM		159	84.45	32.50		
		OI-DRGs-LM		176	85.47	39.21		
			FSS-LH	37	114.05	49.07	2.511	0.085
			TI-DRGs-LH	46	101.76	32.40		
			OI-DRGs-LH ⑤	39	95.07	29.25		
Intraoperative blood loss (ml)	FSS			196	65.79	123.48	1.972	0.140
	TI-DRGs			205	49.68	95.86		
	OI-DRGs			215	46.51	92.90		
		FSS-LM		159	57.52	103.54	0.695	0.500
		TI-DRGs-LM		159	45.31	75.45		
		OI-DRGs-LM		176	49.43	99.94		
			FSS-LH	37	101.35	184.20	2.301	0.105
			TI-DRGs-LH	46	64.78	146.19		
			OI-DRGs-LH ⑤	39	33.33	48.89		

Continuous variables across multiple groups were assessed using analysis of variance (ANOVA), followed by the least significant difference (LSD) test for pairwise comparisons. FSS: Fee-for-service. TI-DRGs: Trial implementation of the diagnosis-related group payment system; OI-DRGS: Official implementation of the diagnosis-related group payment system; LH: Laparoscopic hysterectomy; LM: Laparoscopic myomectomy. ① LSD pairwise comparison indicated a significant difference compared with the FSS group. ② LSD pairwise comparison indicated a significant difference compared with the TI-DRGs group. ③ LSD pairwise comparison indicated a significant difference compared with the FSS-LM group. ④ LSD pairwise comparison indicated a significant difference compared with the TI-DRGs-LM group. ⑤ LSD pairwise comparison indicated a significant difference compared with the FSS-LH group. ⑥ LSD pairwise comparison indicated a significant difference compared with the TI-DRGs-LH group.

Table 1 also presents data on patient age, length of hospital stay, operation duration, and intraoperative blood loss across the three time periods. No significant differences were observed in these parameters for the overall cohort. Similarly, no significant differences were found among patients who underwent laparoscopic myomectomy across the three groups. However, for patients who underwent laparoscopic hysterectomy, significant differences were noted in both operation duration and intraoperative blood loss between the FFS-LH and OI-DRGs-LH groups. Specifically, the OI-DRGs-LH group had a shorter average operation time (95.07 min vs. 114.05 min) and lower intraoperative blood loss (33.33 mL vs. 101.35 mL).

Table 2 summarizes data on postoperative anemia and blood transfusion rates. According to Chinese clinical guidelines, anemia in non-pregnant women is defined as a hemoglobin level below 110 g/L. All patients underwent preoperative and postoperative (day 2) complete blood cell count testing at our institution. During the FFS period, 57 patients (29.08%) developed postoperative anemia, including 48 in the FFS-LM subgroup and 8 in the FFS-LH subgroup. In the TI-DRGs group, 52 patients (25.37%) developed postoperative anemia (44 in the TI-DRGs-LM subgroup and 8 in the TI-DRGs-LH subgroup). In the OI-DRGs group, 74 patients (38.07%) developed postoperative anemia, with 67 in the OI-DRGs-LM subgroup and 7 in the OI-DRGs-LH subgroup. Blood transfusion rates were comparable across all groups: 14 patients (7.14%) in the FFS group, 16 patients (7.80%) in the TI-DRGs group, and 16 patients (7.44%) in the OI-DRGs group received transfusions. There were no significant differences in transfusion rates among the groups or their subgroups.

**Table 2:** Descriptive analysis of basic characteristics (categorical variables).

Group	LM-related subgroup	LH-related subgroup	Patients with postoperative anemia (*n*)	Patients without postoperative anemia (*n*)	X^2^	*P*-value	Patients with blood transfusion (*n*)	Patients without blood transfusion (*n*)	X^2^	*P*-value
FSS			57	196	4.172	0.124	14	182	0.064	0.969
TI-DRGs			52	205			16	189		
OI-DRGs			74	215			16	199		
	FSS-LM		49	110	4.386	0.112	10	149	0.858	0.651
	TI-DRGs-LM		44	115			10	149		
	OI-DRGs-LM		67	109			15	161		
		FSS-LH	8	29	0.270	0.874	4	33	3.034	0.219
		TI-DRGs-LH	8	38			6	40		
		OI-DRGs-LH	7	32			1	38		

Postoperative anemia was defined as hemoglobin (Hb) < 110 g/L. Categorical variables were analyzed using the Pearson chi-square test. FSS: Fee-for-service. TI-DRGs: Trial implementation of the diagnosis-related group payment system. OI-DRGS: Official implementation of the diagnosis-related group payment system; LH: Laparoscopic hysterectomy; LM: Laparoscopic myomectomy.

No deaths or hospital readmissions were reported in any of the three groups during the 6-month follow-up period. After log transformation, total medical costs exhibited a normal distribution. A multiple linear regression model was developed to predict log-transformed total medical costs and assess the impact of DRG period, age, length of hospital stay, type of surgery, number of concurrent procedures, and presence of comorbidities (Table 3). Consistent with earlier findings, total medical costs were not significantly associated with age (*p* = 0.253) or comorbidities (*p* = 0.857). However, there was a significant positive correlation between the DRG period and total medical costs (*p* < 0.001). After adjusting for covariates, the DRG period was associated with a 0.023 increase in log-transformed total medical costs (*p* < 0.001), corresponding to a clinically meaningful cost increase of 2.3–4.7%.

**Table 3:** Multiple regression model for log-transformed total medical costs.

Predictor	Coefficient (B)	Standard error	Standardized Coefficient (β)	*t*-value	*P*-value	Tolerance	VIF
Intercept (constant)	8.999	0.034		263.437	<0.001		
DRGs period	0.023	0.006	0.116	4.181	<0.001	0.965	1.036
Age (years)	−0.001	0.001	−0.035	−1.145	0.253	0.783	1.277
Length of stay (days)	0.036	0.003	0.336	12.187	<0.001	0.971	1.030
Operation type	0.122	0.013	0.295	9.750	<0.001	0.809	1.237
Number of concurrent surgeries	0.088	0.004	0.557	20.139	<0.001	0.965	1.036
Comorbidities	−0.002	0.011	−0.005	−0.180	0.857	0.947	1.056

R^2^ = 0.550; Adjusted R^2^ = 0.545; Standard error of the estimate = 0.111; ANOVA: F = 123.897; DRGs period: FSS = 1, TI-DRGs = 2, OI-DRGs = 3; Operation type: LM = 1, LH = 2; Comorbidities: Presence of comorbidities = 1, No comorbidities = 0. FSS: Fee-for-service. TI-DRGs: Trial implementation of the diagnosis-related group payment system; OI-DRGS: Official implementation of the diagnosis-related group payment system; LH: Laparoscopic hysterectomy; LM: Laparoscopic myomectomy; VIF: Variance Inflation Factor.

## Discussion

### Global implementation of DRG systems and their impact on health care delivery

In July 2024, China’s National Health Insurance Bureau released its latest standard for DRGs. This initiative aims to control healthcare costs and regulate diagnosis and treatment practices. Originally introduced in the United States as a cost-containment strategy, the DRG-based payment system has since been adopted and adapted by numerous countries to fit their healthcare models. In the United Kingdom, France, and Germany, DRG-based payment systems have been credited with improving hospital efficiency, increasing transparency, reducing waiting times and lengths of stay, enhancing care quality, and promoting competition among hospitals. Similarly, in Sweden and Finland, DRG classifications have been widely used for health planning and management, significantly enhancing transparency and operational efficiency in hospital service delivery (10, 11). Robert A. Bergson (12) highlighted that DRG-based payment systems can incentivize healthcare providers, reduce unnecessary services, and promote intra-organizational collaboration, thereby improving both quality and efficiency. In South Korea, Kwak et al. (13) reported that the adoption of DRGs for adenotonsillectomy and tonsillectomy procedures led to lower medical costs without significantly affecting hospital stay length or postoperative complication rates.

However, the effectiveness of DRG systems remains a subject of debate. Several studies have shown limited impact on medical efficiency and quality. For example, Louis et al. (14) systematically analyzed discharge records from nine disease categories across 32 medical institutions in Italy and found that although inpatient volume decreased following the implementation of DRG-based payments, there were no significant changes in patient mortality or readmission rates. Similarly, Chok et al. (6) examined national health insurance data from all hospitals in Switzerland between 2009 and 2013 and concluded that DRG implementation had no significant effect on mortality rates or the duration of intensive care stays among critically ill patients. In South Korea, Kwak et al. (13) conducted a study involving 1,402 patients and found no notable change in the average length of hospital stay after the introduction of DRG payment. Moreover, Sari et al. (15) and Vuagnat et al. (16), using regression models with regional insurance data, reported a significant increase in hospital readmission rates following DRG reform, contrary to the system’s intended goal of improving care quality. Kim et al. (17) attributed rising total hospitalization costs to an unreasonable increase in reimbursement levels under the DRG model. Additional studies by Psaty et al. (18), Silverman et al. (19), and Dafny et al. (20) confirmed varying degrees of “upcoding” across institutions during DRG implementation, where diagnoses were adjusted to increase reimbursement. Furthermore, Kim et al. (17) and Lee et al. (21) employed mathematical models to study hospitals in South Korea adopting DRG reforms and found a rise in both the number and cost of outpatient services during the peri-hospitalization period, suggesting a shift from inpatient to outpatient care in response to DRG-based payment incentives.

### Cost dynamics and service impacts of the DRGs pilot in Zhejiang Province

In Zhejiang Province, a pilot program for DRGs in medical performance management began on October 1, 2021, with official implementation starting on December 1, 2023. This study analyzes three distinct periods: a 6-month fee-for-service (FFS) period, a 6-month trial implementation of DRGs (TI-DRGs) period, and a 6-month official implementation of DRGs (OI-DRGs). The focus of the analysis is to determine if the implementation of DRGs impacts medical costs, service outcomes, and provider behavior at our center. Our findings show no significant difference in total costs during the TI-DRGs period compared to the FFS period. However, there was a noticeable increase in costs during the OI-DRGs period, particularly in operation and examination costs. Specifically, the cost of laparoscopic myomectomy increased during the OI-DRGs period, which likely contributed to the higher overall and surgical costs. Conversely, drug and supply costs slightly decreased during the OI-DRGs period. When examining subgroups, costs during the TI-DRGs-LM period were lower than those during the FFS-LM period across all categories. However, costs during the OI-DRGs-LM period were higher than those during both the FFS-LM and TI-DRGs-LM periods. In contrast, the costs associated with laparoscopic hysterectomy did not show a statistically significant difference.

These findings suggest that, initially, DRGs implementation led to a decrease in the costs of laparoscopic myomectomy, but over time, these costs increased. In the field of general surgery, Kim et al. (22) found that DRG implementation for appendectomy did not significantly impact medical costs, aligning with our findings during the trial period. However, they observed a reduction in hospital stay length. Meng et al. (23) indicated that while DRG-based payment have the potential to reduce costs by shortening the length of stay (LOS), they may also be linked to higher readmission rates. Other studies have similarly highlighted the benefits of DRGs, including reduced healthcare costs and shorter LOS (24).

### Unexpected outcomes of DRG adoption in our hospital setting

In our study, we found no significant differences in LOS, operation duration, or intraoperative blood loss across the three periods. This raises an important question: why did medical costs increase following DRG implementation, even though LOS remained unchanged at our center? A previous study (25) also demonstrated that DRG-based payments initially reduced LOS, but the effect tended to stabilize over time. It is not uncommon for new systems to be actively embraced at first and gradually receive less attention as time goes on. Another factor could be market competition. In a competitive environment, healthcare providers may increase the volume and complexity of medical services to maintain or enhance profit margins. Our study observed a high rate of concurrent surgeries, such as ovarian cyst removal, oviduct cyst removal, and hysteroscopy combined with laparoscopic myomectomy or hysterectomy, after DRG implementation. While concurrent surgeries inherently increase LOS and total costs, current DRG reimbursement often fails to account for these expenses. Nevertheless, clinically justified concurrent surgeries provide important patient benefits by reducing the need for multiple operations. Finally, after healthcare providers adapted to the TI-DRGs period, the increase in surgical costs during the OI-DRGs period may be partly explained by practices such as diagnostic upcoding and varying degrees of coding escalation. Prospective audits comparing coded diagnoses with actual surgical findings could help distinguish legitimate case-mix adjustments from reimbursement optimization strategies.

Our statistical analysis revealed that the proportions of patients undergoing two or more concurrent surgeries were 69.4% in the FFS group, 64.4% in the TI-DRGs group, and 90.2% in the OI-DRGs group. Similarly, the prevalence rates of preoperative systemic comorbidities, such as diabetes, hypertension, and thyroid disorders, were 70.4, 68.3, and 85.6%, respectively. Following the trial implementation of DRGs-based payments, lower-tier hospitals likely recognized that treating patients requiring concurrent surgeries or those with multiple comorbidities could result in financial losses under the DRG reimbursement model. As a result, these more complex cases were increasingly referred to higher-tier hospitals like ours. This referral trend led to a notable rise in the number of medically complex patients, defined by the presence of both concurrent surgeries and comorbidities, during the OI-DRGs period. Ultimately, this shift contributed to a significant increase in surgical and examination costs, as additional diagnostic efforts were undertaken to manage perioperative risks.

### Strategic DRG optimization for a value-based healthcare future

The expansion or implementation of new reimbursement systems such as DRGs must be approached with caution. First and foremost, an appropriate set of indicators for evaluating medical quality under the DRG system should be established. Prioritizing DRG adoption at the expense of care quality and patient safety is misguided. For instance, Meng et al. (23) found that while DRG-based payments can reduce costs by shortening the LOS, this may also associated with higher readmission rates. Therefore, policymakers considering DRG-based payment systems should closely monitor hospital readmission rates in comparison to those under cost-based systems. Given the Chinese government’s strong commitment to value-based healthcare, there is growing emphasis on the quality and value of inpatient services delivered by hospitals (26). Thus, healthcare capacity, efficiency, and service quality should be regarded as core indicators in achieving value-based healthcare.

However, it is important to recognize that the effects of DRG implementation can be highly context-specific, influenced by local policies, hospital practices, and patient case-mix. These factors may limit the generalizability of our findings beyond this hospital or region.

To optimize the DRG system, we offer several recommendations. First, it is essential to reduce unnecessary testing and medication use, as well as to limit the reliance on disposable medical supplies. Concurrent surgeries should be performed judiciously, only when medically appropriate, to avoid inflating costs without added clinical benefit. Second, the training of medical staff should be strengthened. Under the DRG system, patients have greater flexibility in choosing hospitals without being constrained by cost, making service quality a key factor in a hospital’s competitiveness. Third, a triage system should be developed to prioritize emergency cases, enhance operational efficiency, and reduce the incidence of unnecessary surgical procedures. Although the new payment model is expected to drive significant changes to the healthcare system, its implementation and impact must be evaluated in terms of each country’s unique healthcare environment. Our study was conducted at a single center with a relatively small sample size, did not include a detailed analysis of potential issues related to DRG payment, such as preferential admission of less severe cases, the transfer of critically ill patients, diagnostic upcoding, or under-provision of services. To address these limitations and gain more comprehensive insights, we recommend future multi-center studies with larger sample sizes.

## Conclusion

In summary, the implementation of DRGs for laparoscopic uterine leiomyoma surgery did not lead to a significant reduction in total medical costs. Overall costs were influenced by multiple factors, including the DRG phase, length of stay, type of surgery, and the presence of concurrent procedures. The findings from our single-center study differ from the mainstream view, highlighting that the effects of DRG implementation can be highly context-specific, shaped by local policies, hospital practices, and patient case-mix, which may limit the generalizability of these results beyond our institution or region. To effectively reduce total medical costs associated with laparoscopic uterine leiomyoma surgery, we recommend that cost projections be grounded in local empirical data, including detailed clinical records, itemized billing information, and rigorous patient-mix stratification analyses. In addition, a tailored payment standard should be developed for different patient groups, especially those with comorbidities or requiring concurrent surgeries.

## Data Availability

The raw data supporting the conclusions of this article will be made available by the authors, without undue reservation.
